# Impact of the southern annular mode on extreme changes in indian rainfall during the early 1990s

**DOI:** 10.1038/s41598-021-82558-w

**Published:** 2021-02-02

**Authors:** Pao-Wei Huang, Yong-Fu Lin, Chau-Ron Wu

**Affiliations:** 1 Department of Earth Sciences, National Taiwan Normal University, Taipei, Taiwan; 2 Department of Earth System Science, University of California at Irvine, Irvine, CA USA

**Keywords:** Physical oceanography, Climate-change impacts, Physical oceanography

## Abstract

The variability in rainfall amounts in India draws much attention because it strongly influences the country’s ecological and social systems. Indian rainfall is associated with climate factors, including El Niño/Southern Oscillation and the Indian Ocean Dipole. Here we identified the Southern Annular Mode (SAM), the primary pattern of climate variability in the Southern Hemisphere, as the ultimate forcing leading to decadal changes in Indian rainfall. Through statistical analyses using observational data covering the period from 1979 to 2015, we show an increase in the decadal rainfall amount in the early 1990s over the Indian region. Examining atmospheric environmental conditions, we demonstrate that conditions have become more favorable over the past few decades. Specifically, during the positive SAM phase since the early 1990s, changes in the atmospheric fields have evoked anomalous vertical motion over the continent and the Indian Ocean, enhancing the southerly cross-equatorial flow by increased land–sea thermal contrast, thereby increasing decadal rainfall in the region.

## Introduction

Up to 80% of the total annual rainfall over India^1^, a densely populated country, is modulated by the South-Asian summer monsoon, a part of the Asian summer monsoon. Spatial and temporal changes in the amounts of annual rainfall in this area have had sweeping impacts on agriculture, water resources, health, and food security^2^. With more accurate prediction of Indian rainfall events, the numerous effects of weather on the economy, fishery industries, disaster aid, and society can be ameliorated.

Although Indian rainfall is characterized by a significant seasonal cycle, the intensity and magnitude of rainfall events indicate interannual variability related to specified climate factors, such as El Niño/Southern Oscillation (ENSO) and the Indian Ocean Dipole (IOD)^3–5^. For example, Goswami and Xavier^6^ found a negative correlation between Indian rainfall and ENSO, showing that ENSO decreased the meridional gradient of tropospheric temperature over the monsoonal region and shortened the rainy season, resulting in reduced rainfall. However, Saji et al.^7^ pointed out that the IOD may interfere with the influence of ENSO on the southern Asian climate. Abram et al.^8^ reconstructed IOD data using coral oxygen isotope records and demonstrated an increase in the recent IOD, which was fed back to the Indian monsoon.

In addition to Indian climate, variability in Indian monsoon rainfall appears to respond to decadal changes in the sea surface temperature (SST) of the Pacific Ocean^9^. Several studies have shed important light on variability in Indian rainfall in response to the imposed forcing in the Pacific. The Pacific Decadal Oscillation (PDO) is the dominant mode of decadal variability across the tropics and middle latitudes. However, the PDO and ENSO induce this variability on different time scales, although their SST patterns are rather similar. Krishnamurthy and Krishnamurthy^10^ showed that during the PDO positive phase and El Niño period, the anomalous Walker circulation affects local Hadley cell enhancement and impedes the organization of precipitation in India.

Furthermore, several recent studies have indicated that climate factors in the Southern Hemisphere are capable of impacting the climatic characteristics of the Northern Hemisphere^11^. In particular, many regional climates in the Northern Hemisphere, such as in Australia, Asia, and North America, are closely related to the Southern Annular Mode (SAM)^12–14^. The SAM index is used to calculate the difference in the zonal mean sea level pressure anomaly between 40 and 65°S and is considered the dominant mode of variability in the middle and high latitudes of the Southern Hemisphere^15^. In this study, the ultimate forcing leading to decadal changes in rainfall amounts in India was examined; the decadal variability in the SAM is proposed as the likely candidate.

## Decadal variability in Indian rainfall

**Figure 1:**
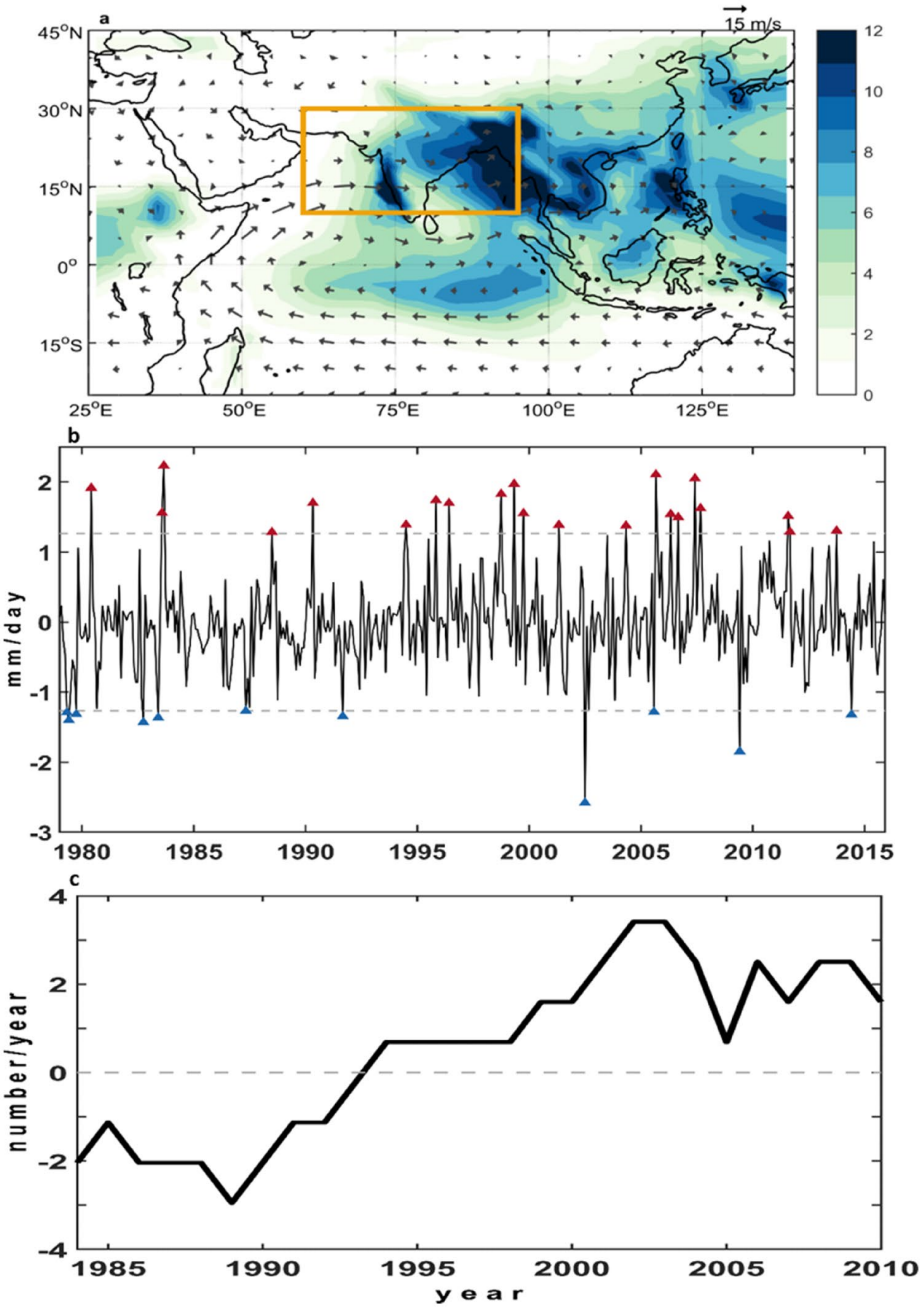
Indian rainfall anomalies and extreme rainfall events. (**a**) Precipitation (shading) and the horizontal wind (vectors) at 850 hPa in boreal summer (JJAS) averaged over 1979–2015. Orange rectangle (10°N–30°N and 60°E–95°E) indicates the study area. (**b**) Time series of monthly Indian rainfall anomaly averaged over the Indian region during 1979–2015. Dashed lines denote 2 standard deviations. Red and blue triangles point out extreme rainfall events and drought events, respectively. (**c**) Time series of extreme rainfall events, which exceed 2 standard deviations with 11-year running mean. The figures are generated with Matlab (R2015a; https://www.mathworks.com/).

Figure 1a shows the annual average rainfall and low-level winds at 850 hPa in boreal summer (June–September) in the Asian monsoon region from 1979 to 2015. The prevailing horizontal wind velocity clearly changes direction to southwesterly in the Northern Hemisphere from southeasterly in the Southern Hemisphere around the tropical Indian Ocean. The rainfall pattern of the Indian region tends to be inhomogeneous and is generally heavier on land than in the ocean.

Figure 1b depicts monthly Indian rainfall anomalies averaged over the domain between 10°N–30°N and 60°E–95°E (the orange rectangle in Fig. 1a, according to Li et al.^16^). The 37-year time series reveals apparent extreme circumstances alternating between rainy and arid periods. More specifically, the number of extreme rain events has increased considerably over the past few decades, which agrees with previous findings that these events are happening with increasing frequency in the Indian region during monsoon season^17^. Hence, Indian rainfall involves not only interannual variability but also decadal variability.

To better describe the long-term patterns in Indian rainfall, we applied an 11-year running mean to a time series of extreme rain events. Figure 1c shows a conspicuous increase during this period, in particular from late 1980 to early 2010, revealing the decadal variability in Indian rainfall. Deka et al.^18^ suggested that the increase in rainfall variability in recent decades is capable of increasing the uncertainty of flood and drought events in the Indian region. Unlike previous studies that focused only on summer rainfall, the analyses of year-round precipitation data in the current study reveal the occurrence of extreme rainfall in not only boreal summer but also the ensuing months, in particular since 1993, as shown in Table S1. Several studies pointed out that Asian monsoon has undergone climate shifts in recent decades, following 1976/77 PDO regime shift^19^ and since the mid-to-late 1990s^20,21^. Nevertheless, the year 1993 coincides with the recent phase change in the SAM index, which implies its potential impact on extreme Indian rainfall. Nevertheless, the dominant climate factor leading to decadal variability in Indian rainfall requires closer examination.

The relative importance of climate factors, including ENSO, the IOD, the PDO, and the SAM, can be quantified through multiple regression analyses. Because the SAM is characterized by both interannual variability^22^ and decadal variability^23^, the regression employed in the SAM was performed separately on interannual and decadal time scales. The regression coefficient revealed that decadal variability in the SAM has an advantage over the other indices, with statistical significance above the 99% confidence level. Although both ENSO and the IOD are statistically significant, their main signal is characterized by interannual variability rather than decadal variability.

**Figure 2:**
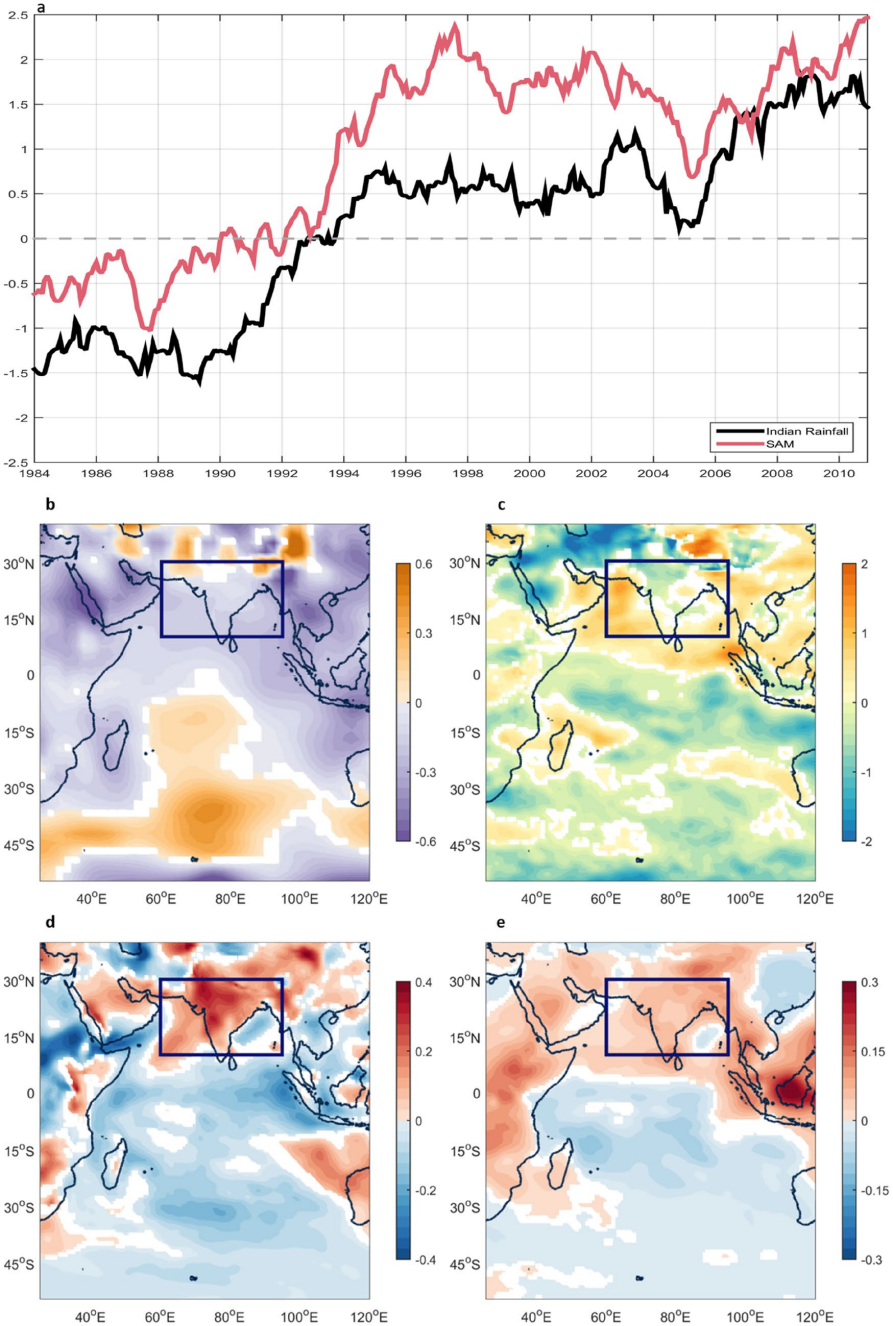
SAM indices and atmospheric conditions. (**a**) Monthly time series of the SAM index (red curve) and Indian rainfall (black curve) with 121-month running mean and normalized during 1979–2015. Regression pattern of (**b**) mean sea level pressure (hPa); (**c**) potential vorticity (10^−8^ K m^2^/kg s) at 500 hPa; (**d**) specific humidity (g/kg) at 850 hPa; (**e**) specific humidity (g/kg) at 500 hPa with 121-month running mean of the SAM index. Colored indicates statistical significance above the 99% confidence level. The figures are generated with Matlab (R2015a; https://www.mathworks.com/).

Figure 2a shows a monthly time series of normalized Indian rainfall anomalies (black curve) along with normalized decadal SAM indices (red curve) during the period from 1979 to 2015; a 121-month running mean was applied to both time series. The two time series show close agreement, with a correlation coefficient of 0.91, which is above the 99% significance level. The high correlation suggests the substantial impact of the decadal SAM on Indian rainfall. The SAM and Indian rainfall have similar variability during the study period, with a negative phase before 1993 that changes to a positive phase and increases considerably thereafter. Both likely have a long-term upward trend over the past three decades, with the exception of the time period around 2005.

Figure 2b–e show the atmospheric conditions affected by the SAM, including the dynamic conditions of mean sea level pressure and potential vorticity, as well as the thermodynamic condition of specific humidity, all of which are projected by the SAM (single regression analyses). Terrestrial land adjacent to the northern Indian Ocean has a slightly anomalous low, whereas an anomalous high extends from 60 to 85°E in longitude and from 5 to 50°S in latitude in the central basin (Fig. 2b). The meridional pressure gradient pattern, therefore, can create convergence from the central Indian Ocean to the study area at the low level, also advantageously impacting the organization of the convective system. Figure 2c shows potential vorticity at mid-level (500 hPa), with positive anomalies in the study area but negative ones over the rest of the Indian Ocean. In general, anomalous positive potential vorticities at mid-level are conducive to the formation of convective instability, whereas anomalous negative values are unfavorable for convection.

In addition to the enhanced meridional pressure gradient and increased potential vorticity of the dynamic conditions, the thermodynamic conditions are also performed in this study. Figure 2d–e show regression results for specific humidity at 850 hPa and 500 hPa, respectively; spatially coherent anomalous wet conditions are shown in the study area, whereas the Indian Ocean, in particular south of the equator, displays anomalous dry conditions. This implies a large-scale vertical environment filled entirely with moisture over the study area. The conditions are supportive of stronger convective activity, in particular in the study area. These results also imply that the SAM alters large-scale dynamics and thermodynamics to produce favorable conditions for rainfall over the study area on a decadal time scale.

## Mechanism

**Figure 3:**
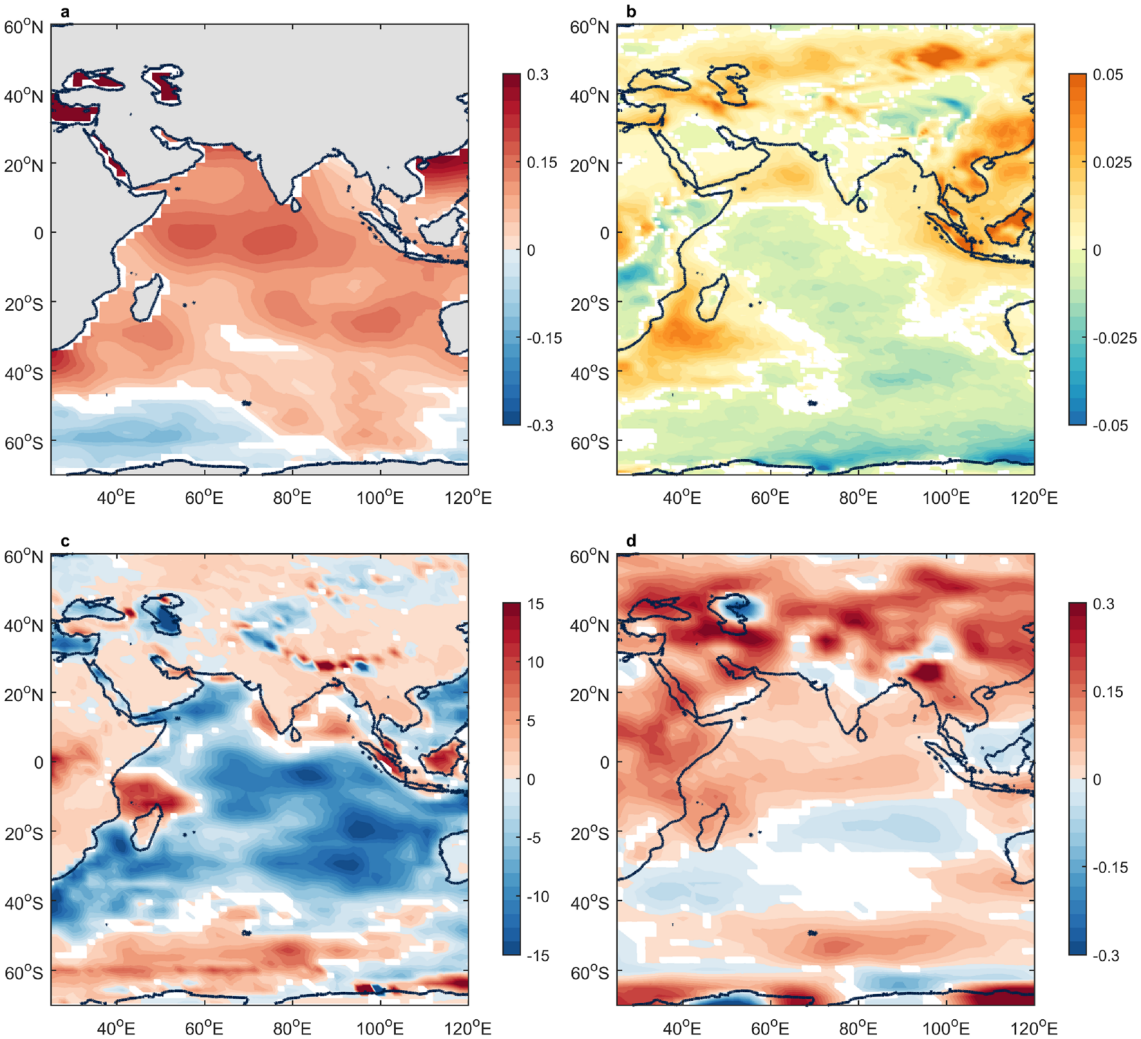
Regression patterns. (**a**) Sea surface temperature (°C); (**b**) low-level cloud cover (%); (**c**) net heat flux (W/m^2^); (**d**) surface air temperature (K) with 121-month running mean of the SAM index. Colored indicates statistical significance above the 99% confidence level. The figures are generated with Matlab (R2015a; https://www.mathworks.com/).

The trans-hemisphere influence of the SAM on decadal variability in Indian rainfall can be explained by cross-equatorial circulation. During the positive SAM phase since the early 1990s, the SST anomaly over the Southern Ocean has shown anomalous warm (cool) conditions in the subtropics (extratropics), as shown in Fig. 3a. The anomalous ascending and descending motions are commonly accompanied by anomalous SSTs over the subtropical and extratropical regions, respectively. The change in the vertical motion weakens Ferrel and Hadley cells in the Southern Hemisphere by offsetting the general circulation. Simultaneously, adjacent meridional circulations of the Hadley cell in the Northern Hemisphere adjust and weaken^24^. Associated with changes in vertical motion, the strong positive anomalous low-level cloud cover (LCC) extends to the eastern coast of South Asia and the East Asian continent, whereas the Indian Ocean (0–50°S and 55–95°E) shows a negative anomalous LCC (Fig. 3b). LCC has a large impact on the surface net heat flux (NHF). A higher LCC generally traps more radiation, which is released from the surface. Figure 3c also shows that a positive anomalous NHF (in which the Earth’s surface gains heat) prevails in the Asian continent, whereas an obviously negative anomalous NHF (in which the Earth’s surface releases heat) is commonly found over the Indian Ocean. Accordingly, the surface air temperature (SAT; Fig. 3d) exhibits a substantially strong positive anomaly across the Asian continent, including South Asia and East Asia, whereas only a slightly positive anomalous SAT or even a negative anomalous SAT appears over almost the entire Indian Ocean.

**Figure 4:**
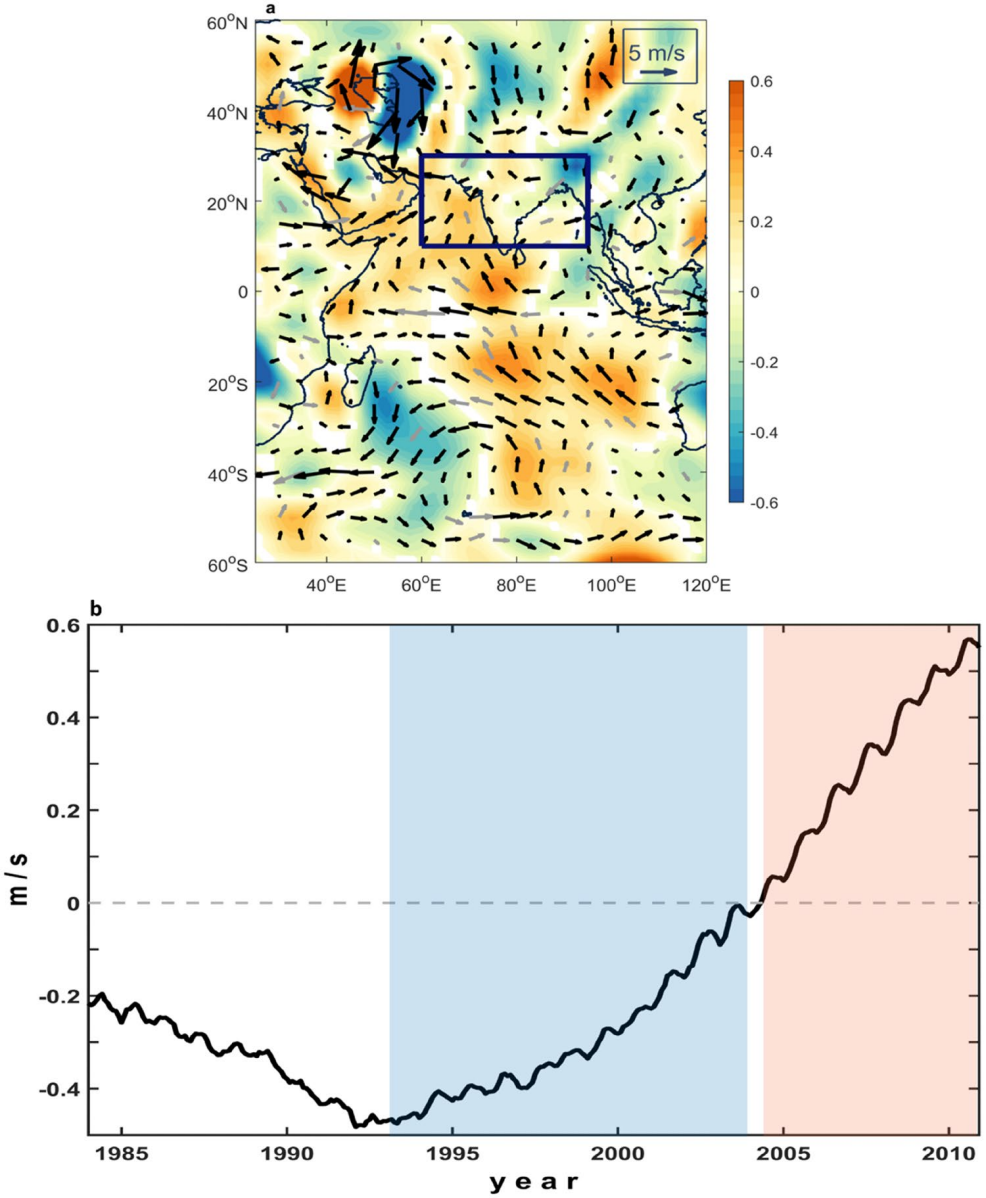
Meridional wind. (**a**) Regression pattern of meridional wind (shading) as well as the horizontal wind (vectors) at 850 hPa with 121-month running mean of the SAM index. Colored and black vectors indicate statistical significance above the 99% confidence level. (**b**) Time series of meridional wind speed anomaly at 850 hPa with 121-month running mean averaged over the equatorial Indian Ocean (55°E–85°E and 5°S–5°N) during 1979–2015. Positive and negative values represent northerly and southerly winds, respectively. The figures are generated with Matlab (R2015a; https://www.mathworks.com/).

The SAT pattern indicates a remarkable land–sea thermal contrast between the Indian Ocean and Asian continent that induces a southerly cross-equatorial flow over the Indian Ocean. Consequently, the pronounced intensification of the southerly cross-equatorial flow in the Indian Ocean leads to increased rainfall, as shown in Fig. 4a in the regression pattern of meridional wind speed at 850 hPa. For the sake of explicitness, we further quantified meridional wind speed anomalies averaged over the equatorial Indian Ocean (5°S–5°N and 55–85°E). Figure 4b shows a monthly time series of the anomalies at 850 hPa with a 121-month running mean during the period from 1979 to 2015. The time series reveals a prominent rise beginning in the early 1990s, when the SAM shifted to its positive phase; this indicates that the anomalous southerly wind was gradually enhanced. During the positive SAM phase, the meridional wind speed changed slightly beginning around 1993 (blue shading) accompanied by a slight change in rainfall, whereas it became more pronounced after 2004 (red shading), leading to increased rainfall, which bears out the impact of the decadal SAM on the variability in Indian rainfall.

Although the regression patterns are sufficient to explain the linear pattern effected by the SAM, the issue arises as to whether the manifested impact is associated with natural variability in the climate. To address this issue, we used differences between the positive and negative SAM phases for all parameters of this study (both dynamic and thermodynamic) over the period from 1979 to 2015 (Figures S1 and S2). The composite results are consistent with the results of the linear regression, indicating that the SAM is the dominant climate factor over the Indian region on the decadal time scale.

## Summary

The current study demonstrates trans-hemisphere influences on variability in Indian rainfall from the middle and high latitudes of the Southern Hemisphere. Our results indicate that an increase in the SAM is the ultimate forcing leading to significantly enhanced Indian rainfall over the past few decades. In the early 1990s, a positive SAM phase resulted in anomalous warming of the SST in the subtropics and anomalous cooling in the extratropics, offsetting the general circulation and weakening Ferrel and Hadley cells in the Southern Hemisphere. A weaker Hadley cell in the Southern Hemisphere is usually accompanied by a similar weakening of the Hadley cell in the Northern Hemisphere. Consequential vertical motion led to positive anomalous LCC and NHF on the continent but negative anomalies within the Indian Ocean, giving rise to an enhanced land–sea thermal contrast. The increased meridional air temperature gradient induced an intensified southerly cross-equatorial flow that was ultimately responsible for the increased Indian rainfall.

## Data and methods

### Sea surface temperature

Monthly SST data are adopted from the improved Extended Reconstructed SST Version 3 (ERSSTv.3b)^25^ distributed by the National Oceanic and Atmospheric Administration’s Earth System Research Laboratory (NOAA/ESRL), with horizontal resolution of 2° × 2° grids during 1979–2015.

### Precipitation

Precipitation data are from the Global Precipitation Climatology Project Version 2.3 (GPCP V2.3, http://www.esrl.noaa.gov/psd/data/gridded/data.gpcp.html) monthly precipitation product, which is based on a blend of satellite and in-situ measurements since 1979 on global grids of 2.5° × 2.5°.

### Reanalysis data

Monthly atmospheric datasets employed in this study for the period from 1979 to 2015, including meridional wind, zonal wind, downward shortwave radiation, upward solar radiation flux, downward longwave radiation flux, upward longwave radiation flux, sensible heat net flux, latent heat net flux and surface air temperature, are derived from the National Centers for Environmental Prediction (NCEP)/NCAR reanalysis datasets, with horizontal resolution of 2.5° × 2.5°^26^. Additionally, monthly potential vorticity, specific humidity and low clod cover data with horizontal resolution of 0.75° × 0.75° are obtained from European Center for Medium-Range Weather Forecasts (ECMWF) Reanalysis Interim (ERAint) data.

### Climate indices

The Nino-3.4 index (http://www.cpc.ncep.noaa.gov/data/indices/sstoi.indices) is adopted from NOAA to represent the ENSO variability in this study. The Dipole Mode index (DMI) is defined as the SST anomaly difference between the western equatorial Indian Ocean (50–70°E, 10°S–10°N) and the southeastern equatorial Indian Ocean (90°E–110°E, 10°S–Equator)^7^. The PDO index is provided by the JISAO (Joint Institute for the Study of the Atmosphere and Ocean, http://research.jisao.washington.edu/pdo/). The SAM index is defined by Marshall (2003)^27^, which is based on the pressure difference between six stations at 40°S and six stations at 65°S.

### Net heat flux calculation

Net heat flux is the sum of six terms, including downward shortwave radiation (DS), upward solar radiation flux (US), downward longwave radiation flux (DL), upward longwave radiation flux (UL), sensible heat net flux (SH) and latent heat net flux (LH).

$$ {\text{Net heat flux}} = \left( {{\text{DS}} - {\text{US}}} \right) - \left( {{\text{UL}} - {\text{DL}}} \right) - {\text{LH}} - {\text{SH}} $$

### Regression analysis

Multiple linear regression analysis is as follows:

$$ P = \mathop \sum \limits_{1}^{n} \beta_{n } X_{n} + \beta_{0} + \varepsilon $$

where P represents Indian rainfall anomaly in the study area with 5-month running mean, X is climate index, including ENSO, IOD, SAM and PDO. Among them, ENSO and IOD are with 5-month running mean to filter out seasonal signals, while PDO and SAM are with 121-month running mean. All the parameters are normalized by their variance. ε represents unexplained noise. *β*_*n*_ and *β*_0_ are regression coefficients.

Simple linear regression analysis is as follows:

$$ A = \alpha_{1} S + \alpha_{0} + \varepsilon $$

where A represents anomaly of each atmospheric parameter normalized by its variance and with 121-month running mean, S is the SAM index normalized by its variance, ε represents unexplained noise, and *α*_1_ and *α*_0_ are regression coefficients.

## Supplementary Information

Supplementary Information.
