# Supplementary material for: Impact of the southern annular mode on extreme changes in indian rainfall during the early 1990s
Source: Sci Rep. 2021 Feb 2;11:2798. doi: 10.1038/s41598-021-82558-w (PMC7854646; doi:10.1038/s41598-021-82558-w)
Supplement: Supplementary file 1 — Supplementary Information. [file 41598_2021_82558_MOESM1_ESM.docx]

**Impact of the Southern Annular Mode on Extreme Changes in Indian Rainfall during the Early 1990s**

Pao-Wei Huang^1^, Yong-Fu Lin^2^, Chau-Ron Wu^1,*^

^1^Department of Earth Sciences, National Taiwan Normal University, Taiwan

^2^Department of Earth System Science, University of California at Irvine, Irvine, California, USA

*Correspondence to [cwu@ntnu.edu.tw](mailto:cwu@ntnu.edu.tw)

Table S1: Extreme wet and drought events in the Indian region during 1979-2015. Years whose precipitation anomalies exceed 2 standard deviations are in red (see Figure 1b).

| **Year** | **Extreme Rainfall Event** |
| --- | --- |
| **1979-1992** | **1979/05, 1979/06, 1979/10, 1980/06, 1982/10, 1983/06, 1983/08, 1987/05, 1988/07, 1990/05, 1991/09** |
| **1993-2015** | **1994/07, 1995/11, 1996/06, 1998/10, 1999/05, 1999/10, 2001/05, 2002/07, 2004/04, 2005/08, 2005/09, 2006/05, 2006/09, 2007/06, 2007/09, 2009/06, 2011/08, 2011/09, 2013/10, 2014/06** |

**
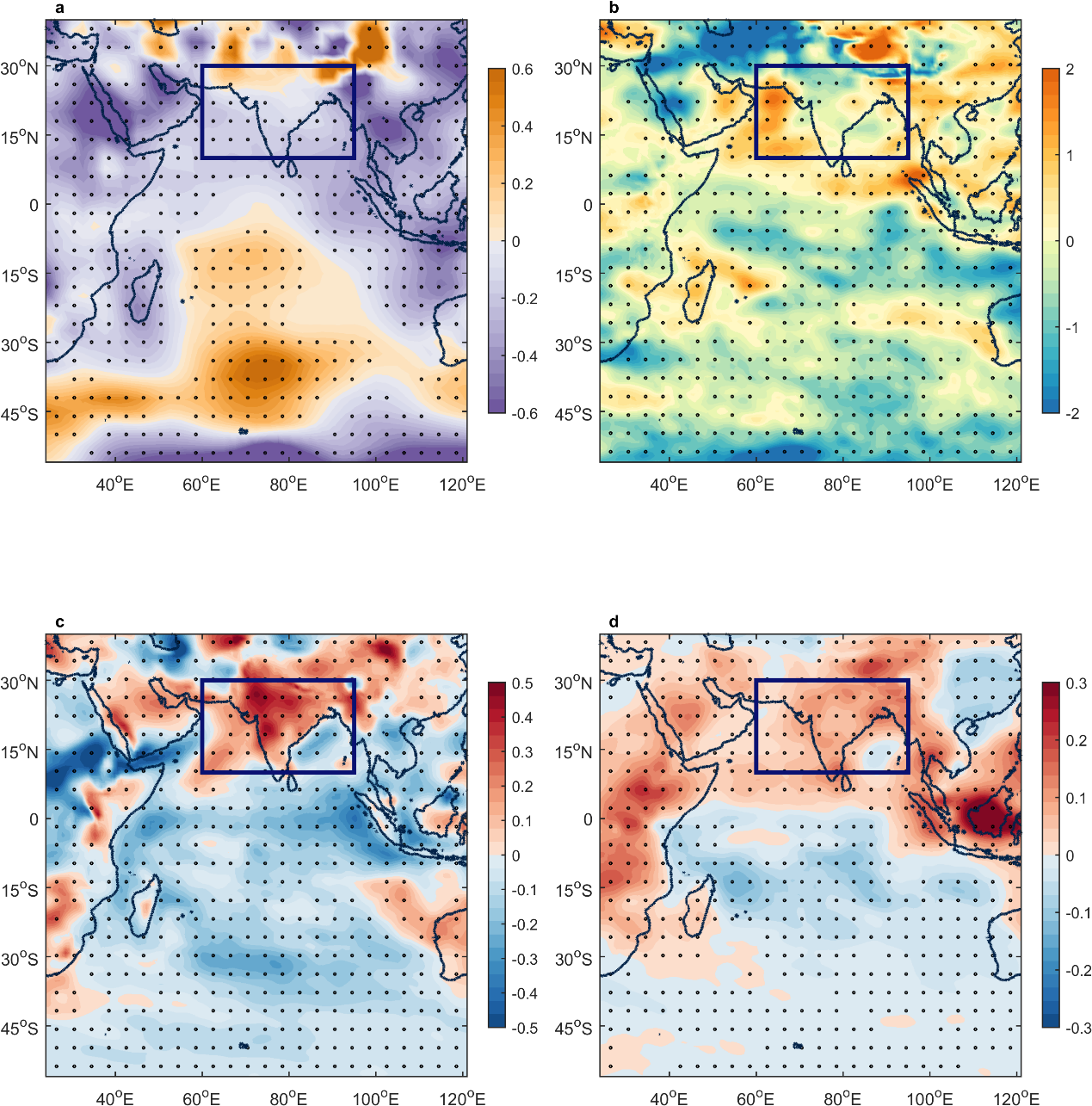
**

Figure S1: Differences between the positive and negative SAM phases during 1979-2015 for (a) mean sea level pressure (hPa); (b) potential vorticity at 500 hPa (10^-8^ K m^2^/kg s); (c) specific humidity at 850 hPa (g/kg); (d) specific humidity at 500 hPa (g/kg). Blue rectangle (10˚N–30˚N and 60˚E–95˚E) indicates the study area. Black dots indicate statistical significance above the 99% confidence level.


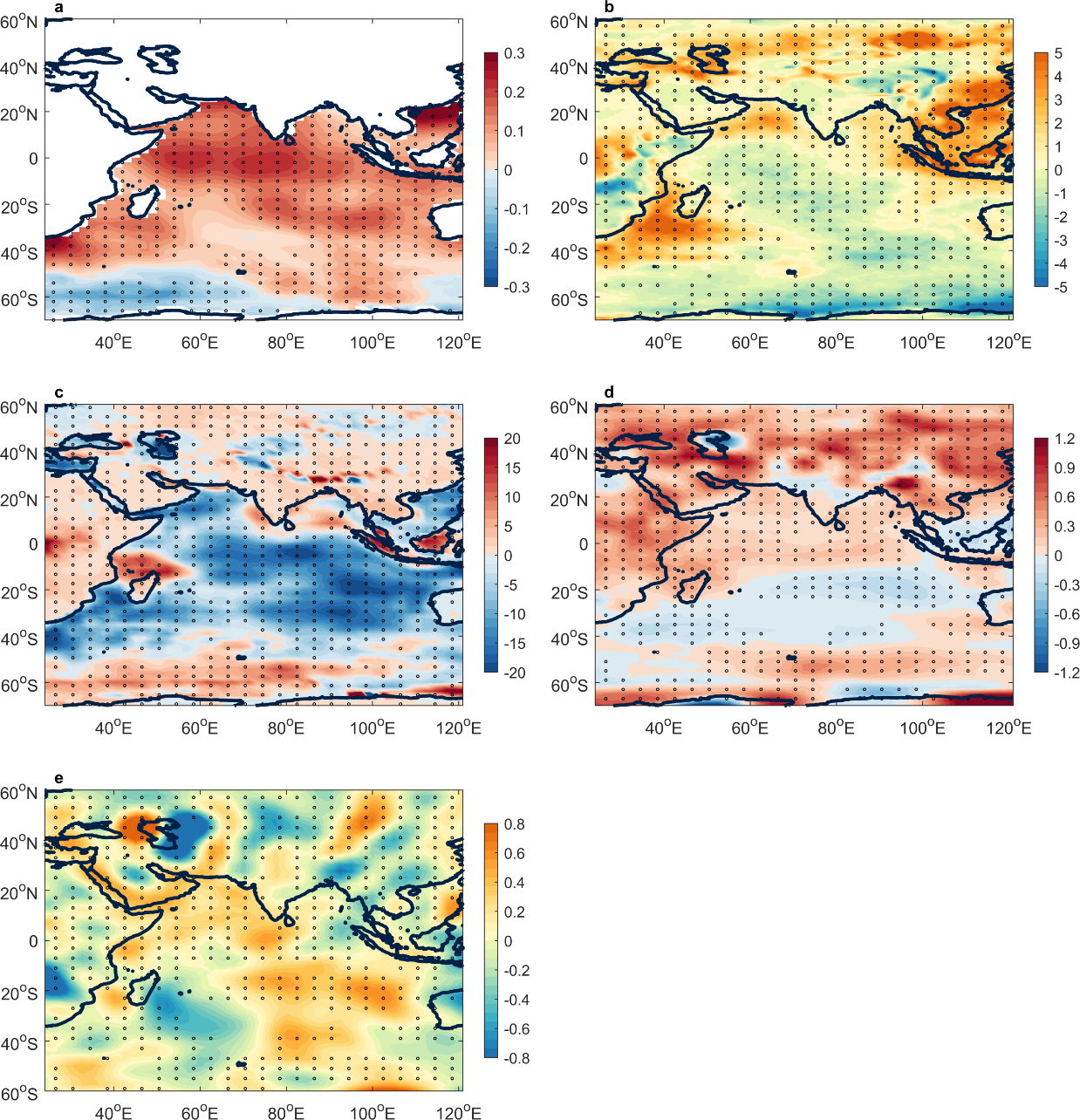


Figure S2: Differences between the positive and negative SAM phases during 1979-2015 for (a) sea surface temperature (⁰C); (b) low-level cloud cover (10^-2^ %); (c) net heat flux (W/m^2^); (d) surface air temperature (K); (e) meridional wind at 850 hPa (m/s). Black dots indicate statistical significance above the 99% confidence level.
